# Engagement of the Mannose Receptor by Tumoral Mucins Activates an Immune Suppressive Phenotype in Human Tumor-Associated Macrophages

**DOI:** 10.1155/2010/547179

**Published:** 2011-02-09

**Authors:** P. Allavena, M. Chieppa, G. Bianchi, G. Solinas, M. Fabbri, G. Laskarin, A. Mantovani

**Affiliations:** ^1^Deptartment Immunology & Inflammation, IRCCS Clinical Institute Humanitas, Rozzano, 20089 Milan, Italy; ^2^Department of Translational Medicine, National Institute of Gastroenterology IRCCS “Saverio de Bellis”, Castellana Grotte, 70013 Bari, Italy; ^3^Advanced Research Centre for Health (ARCHES), Enviroment and Space, Castellana Grotte, 70013 Bari, Italy; ^4^Environmental Health Sciences Department, Mario Negri Institute, 20157 Milano, Italy; ^5^Cardiovascular Research Institute, University of California, San Francisco, CA 94158, USA; ^6^European Commission, Joint Research Centre Institute for Health and Consumer Protection Molecular Biology and Genomics, 21020 (VA) Ispra, Italy; ^7^Department of Physiology and Immunology, University of Rijeka, 51000 Rijeka, Croatia; ^8^Department of Translational Medicine, University of Milan, 20121 Milan, Italy

## Abstract

Tumor-Associated Macrophages (TAMs) are abundantly present in the stroma of solid tumors and modulate several important biological processes, such as neoangiogenesis, cancer cell proliferation and invasion, and suppression of adaptive immune responses. Myeloid C-type lectin receptors (CLRs) constitute a large family of transmembrane carbohydrate-binding receptors that recognize pathogens as well as endogenous glycoproteins. Several lines of evidence demonstrate that some CLRs can inhibit the immune response. In this study we investigated TAM-associated molecules potentially involved in their immune suppressive activity. We found that TAMs isolated from human ovarian carcinoma samples predominantly express the CLRs Dectin-1, MDL-1, MGL, DCIR, and most abundantly the Mannose Receptor (MR). Components of carcinomatous ascites and purified tumoral mucins (CA125 and TAG-72) bound the MR and induced its internalization. MR engagement by tumoral mucins and by an agonist anti-MR antibody modulated cytokine production by TAM toward an immune-suppressive profile: increase of IL-10, absence of IL-12, and decrease of the Th1-attracting chemokine CCL3. This study highlights that tumoral mucin-mediated ligation of the MR on infiltrating TAM may contribute to their immune suppressive phenotype.

## 1. Introduction

Among tumor-infiltrating leukocytes, Tumor-Associated Macrophages (TAMs) constitute a major subset [1–3]. While the presence of T lymphocytes in tumor stroma is usually correlated with more favourable prognosis of cancer patients [4, 5], in most studies the density of TAM is associated with rapid tumor progression [6–9]. TAMs are poorly cytotoxic against neoplastic cells and may actually favour tumor cell survival and proliferation by actively producing growth factors for cancer and endothelial cells. They are also a major source of proteolytic enzymes that degrade the extra-cellular matrix thus favouring the invasion of neoplastic cells [9, 10]. Further, TAM contributes to the evasion of tumors from immune control by producing immune-suppressive cytokines such as IL-10 and TGF*β* [2, 9].

Our group proposed that TAMs are M2-like polarized macrophages [11]. Along a conventional definition, macrophages activated in the presence of inflammatory mediators (e.g., LPS) and Th1 cytokines (e.g., IFN*γ*) are defined as M1 or classically activated macrophages. These effectors have high cytotoxic functions, produce immune-stimulatory cytokines, and are important cells for the defense against intracellular pathogens and transformed cells [11–13]. On the other hand M2 macrophages, or alternatively activated macrophages, differentiate in microenvironments rich in anti-inflammatory mediators or Th2 cytokines (e.g., IL-10, IL-4, IL-13); they have high scavenging activity, produce several growth factors, activating the process of tissue regeneration, and suppress adaptive immune responses [2, 12, 14, 15]. While these activities are of extreme importance during wound healing to return to the homeostatic state, in the context of a growing tumor they are deleterious to the host. Indeed several studies have found a strong correlation between high numbers of TAM, number of vessels, and lower disease-free survival [9, 10, 16–18]. 

In this study we investigated TAM-related mechanisms of immune escape and considered C-type lectin receptors (CLRs) as interesting candidates. CLRs are a large family of structurally related transmembrane receptors which, by virtue of their carbohydrate-recognition domains, bind high affinity sugar moieties present on the surface of pathogens as well as of endogenous glycoproteins [19, 20]. Together with the Toll-like receptor (TLR) family, CLRs expressed on myeloid cells of the innate immunity constitute the major system to sense the outer world [21–23]. Myeloid CLRs are subdivided into two major families: the first including the mannose receptor (MR, DEC206), ENDO180, DEC205, and PLA2 receptor, and the second including DC-SIGN, Dectin-1, Langerin, DCIR, MGL, and BDAC2 [24, 25].

Although the majority of studies have investigated the role of CLRs in the recognition, internalization, and clearance of pathogens through activation of innate immunity, other studies have clearly demonstrated that at least some receptors elicit anti-inflammatory/immune-suppressive responses, raising the hypothesis that pathogens may exploit CLR binding and internalization ability to overcome innate immunity and survive within the host [22, 24, 25]. For instance, M. tuberculosis binds DC-SIGN and the MR and inhibits the production of IL-12 [26–28]. We previously reported in mono-DC that cross-linking of the MR with an agonist antibody increased IL-10 production leading to inhibition of IL-12 and defective Th1 differentiation [27]. Activation of BDAC2, expressed by plasmacytoid DC, as well as DCIR, downregulates the production of IFN*α* [29, 30]. DCIR also interferes with GM-CSF signalling [31]. The receptor MGL recognizes the isoform CD45RB expressed by effector memory T cells and negatively influences T cell receptor signalling [32]. Thus, several lines of evidence point to a role of at least some CLRs in the restriction of inflammatory reactions and in homeostasis preservation [24, 25]. 

Of interest, CLRs recognize glycans expressed also on endogenous ligands. For instance, the carbohydrate sialyl Lewis^X^-type expressed on lymphatic endothelium is recognized by the MR and DC-SIGN [24, 33]. The latter also binds vascular adhesion molecules [23]. The MR recognizes selected hormones (thyroglobulin, luteotropin), matrix molecules (chondroitin sulphate proteoglycans, collagen), and enzymes (myeloperoxidase, lysosomal hydrolases) [20]. DC-SIGN, MGL, and MR bind epithelial mucins [24, 27, 34–36]. The physiological significance of the recognition of endogenous ligands by CLR is not fully characterized.

Previous studies on CLRs have been mainly performed with in vitro generated macrophages and DC or with in vivo mouse models of diseases. The aim of this study is to investigate the expression of CLRs in TAM. Here we show that TAM isolated from human ovarian carcinoma samples predominantly expressed Dectin-1, MDL-1, MGL, DCIR, and most abundantly the MR. Experiments demonstrated that the MR recognizes ligands present in carcinomatous ascites and tumoral mucins such as CA125 and tumor-associated glycoprotein- (TAG-) 72. Upon mucin-engagement of the MR, TAMs secrete higher levels of IL-10 and lower levels of the T cell attracting chemokine CCL3. Thus, tumoral mucin-mediated activation of the MR on TAM triggers an immune-suppressive response which likely contributes to tumor immune evasion.

## 2. Materials and Methods

### 2.1. Isolation of Human Tumor-Associated Macrophages (TAMs)

Having obtained an informed consent, we collected carcinomatous ascites and/or tumor samples from 27 patients with histologically confirmed ovarian tumors. TAMs were isolated from ascites by density Ficoll, and Percoll gradients (Lonza, Italy) as described in [37]; TAMs from solid tumors were isolated by enzymatic digestion and Ficoll gradient [38] and were further purified by plastic adherence (RPM1 1640 w/o FBS, 1h, 37°C); adherent cells were usually 80–90% CD68+ macrophages as assessed by flow cytometry. 

Human in vitro differentiated macrophages were obtained by culture of monocytes with M-CSF (20 ng/mL) [37] for 6 days [37]. Myeloid DCs were differentiated from monocytes with GM-CSF (50 ng/mL) and IL-4 (20 ng/mL) for 6 days [27].

### 2.2. Transcriptional Profile Analysis

TAMs from 7 different patients (5 from carcinomatous ascites and 2 from solid tumors) were used for the transcriptional profiling experiments. TAMs were either immediately processed or after 18-h stimulation with LPS (100 ng/mL) (Sigma, Italy) or IL-10 (20 ng/mL) (Peprotech,Italy) (for 4 TAM preparations). Macrophages from the peritoneal free-fluid of nontumoral patients (ovarian cysts) were collected during surgery from 12 different patients, centrifuged over Ficoll and immediately processed for RNA (purity >90%). Total RNA was extracted from 5 × 10^6^ cells using Trizol (Invitrogen Life Technologies), retrotranscribed and prepared for GeneChip hybridization as previously described [38]. Each TAM preparation was independently tested. Macrophages from nontumoral patients were pooled to reach the minimum necessary amount for 1 GeneChip. Fragmented cRNA was hybridized to HG-U133 Plus 2.0 GeneChips (Affymetrix) and then washed and scanned according to manufacturer's guidelines. Expression measures were computed using Robust Multiarray Average (RMA). Statistical differences were assessed by a moderated *t*-test analysis performed using a Limma bioconductor package, and resulting *P*-values were adjusted using the Benjamini and Hochberg step-up method for controlling the False Discovery Rate (FDR). Genes were defined as regulated when characterized by a fold of induction ≥2 and an FDR *P*-value ≤.05. Computations were conducted using the R statistics programming environment (http://www.r-project.org/).

### 2.3. Phenotype Analysis

Tumor macrophages were analysed by flow cytometry on FACS Canto (BD Bioscience, Milan, Italy). Cells were first incubated with PBS 1% HS (30 minutes 4°C) to block Fc*γ*R, washed and resuspended in FACS buffer (PBS 0.5% BSA, 0.05% NaN_3_). PE-mouse antihuman CD14 (clone M5E2) was purchased from (BD Pharmingen, Italy). Three of mouse anti-human MR/CD206 were used with identical results. Clone 19.2 was from BD Pharmingen; clone PAM-1 was previously characterized [27]; clone WE458 was in-house generated and selected for reactivity against MR-transfected CHO cells.

### 2.4. Endocytosis Assay

Mannose receptor–mediated endocytosis was measured as the cellular uptake of FITC-dextran and quantified by flow cytometry. Approximately 2 × 10^5^ cells per sample were incubated in media containing FITC-dextran (1 mg/mL) (molecular weight 40,000; Sigma) over a period of 60 min. After incubation, cells were washed twice with phosphate-buffered saline (PBS) to remove excess dextran and fixed in cold 1% formalin. Endocytosis was expressed as fluorescence intensity, calculated as mean fluorescence intensity of positive cells at 37°C- mean fluorescence intensity of positive cells at 4°C.

### 2.5. Immunohistochemistry

Human surgical samples of ovarian tumors were immediately frozen in OCT after surgical collection. Sections were stained with anti-CD206 mAbs, followed by a goat antimouse secondary antibody (EnVision horseradish peroxidase rabbit/mouse, DakoCytomation). After a diaminobenzidine reaction (Liquid DAB + Substrate Chromogen System, Dako Cytomation), sections were counterstained with hematoxylin (Mayer, DIAPATH).

### 2.6. Elisa

Cytokines were measured in supernatants of TAM, macrophages, and Dendritic Cells (DCs) by commercially available ELISA kits (IL-10, IL-12, CCL3) according to manufacture's instructions (R&D Systems, Space Import, Milan, Italy). Cells were pretreated (10 min. room temperature) with anti-CD206 (clone WE458, 2 ug/mL), or tumoral mucins (Sigma) TAG-72 (200 UI/mL) or CA125 (200 UI/mL), prior to stimulation with LPS (1 ug/mL, 24 hours). TAG-72 and CA125 contained less than 0.125 endotoxin unit/mL as checked by Limulus amebocyte lysate assay (BioWhittaker, Walkersville, MD).

### 2.7. Statistical Analysis

Prism software (GraphPad) and Microsoft Excel were used for all statistical analyses. Student's *t* tests were used to determine statistically significant differences between experimental groups. *P* < .05 was considered to be statistically significant.

## 3. Results

### 3.1. C-Type Lectin Receptor Gene Expression in Human TAM

To study the expression of CLRs in TAM we interrogated our Affymetrix database performed with 7 different populations of purified TAM isolated from human ovarian carcinoma (5 from carcinomatous ascites and 2 from solid tumors). CLR gene expression levels from TAM of solid tumors or from ascitic fluids were similar and were considered together. The most expressed CLR genes were the mannose receptor (Mrc1, CD206), *Dectin1, DCIR MDL-1,* and *MGL-1* (Table 1). Other CLR genes were expressed at very low level (e.g., *DEC205, DC-SIGN, PLA2R*).

**Figure 1:**
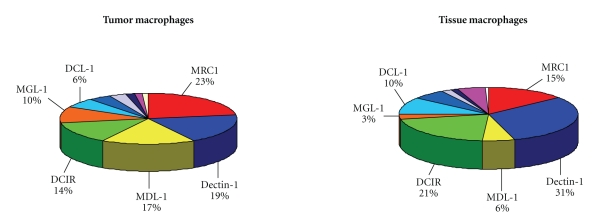
Schematic representation of the relative expression of the eleven CLRs shown in Table 1, in tumoral macrophages (TAM) from ovarian tumor samples and in nontumoral macrophages isolated from the peritoneal free-fluid of patients with benign diseases (ovarian cysts).

Modulation of CLR expression by LPS/IFN*γ* or IL-10 was performed in 4 TAM samples. Exposure of TAM to LPS/IFN*γ* induced a different gene modulation with a prominent increase of DEC205 (2.9-fold) and strong decrease of *Mrc1* (0.1) and of *MDL-1* (0.3). In contrast, pretreatment with IL-10 upregulated *Mrc1* by 1.5-fold (Table 1). It was of interest to compare the gene expression analysis of TAM with normal tissue macrophages. We had the opportunity to test peritoneal free-fluid macrophages collected from nontumoral patients. As the amount of free-fluid and the cellular content is usually very small, we pooled the samples from 12 different subjects who underwent surgery for nonneoplastic diseases (ovarian cysts) and analyzed with the same GeneChips (Affymetrix) used with TAM.  Figure 1 shows that the relative expression of the eleven CLRs analyzed was similar between TAM and normal tissue macrophages, though some differences were noted: *Mrc1*, *MDL-1,* and *MGL-1* were higher in TAM, while *Dectin1* and *DCIR* and *DCL-1* were higher in normal macrophages. 

On the basis of these findings we further investigated the mannose receptor (MR) in human TAM. 

### 3.2. Phenotype and Functional Activity of the MR in Human TAM

**Figure 2:**
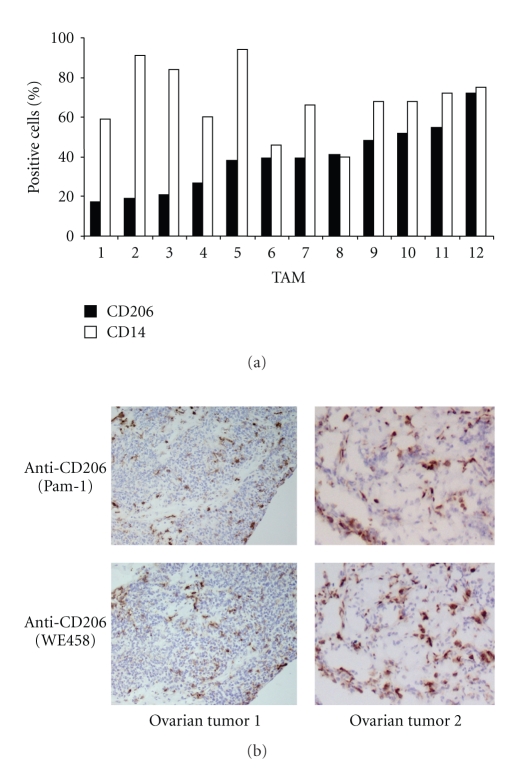
Expression of the Mannose Receptor (MR) by human TAM. (a) Flow cytometry analysis of purified preparations of TAM from carcinoumatous ascites of patients with ovarian cancer. TAMs were stained with anti-CD206 mAb (clone PAM-1) or with anti-CD14. Twelve different preparations were tested. (b) Immunohistochemistry of 2 tumor samples from ovarian cancer tissues stained with anti-CD206 mAbs (clone PAM-1, upper panels; clone WE458, lower panels). Positive cells are brown stained (magnification: left panels 40 x, right panels 100 x).

To check for protein expression, 12 different preparations of human TAM were purified from the ascitic fluid of patients with ovarian carcinoma and tested by flow cytometry.  Figure 2(a) shows the results of each individual preparation as percentage of MR (CD206) and of CD14, used as a pan-myeloid marker. MR expression was variable and ranged from 17% to 72% (median value 39%). Such heterogeneity is likely due to the fact that the MR is an endocytic receptor that continuously shuttles from the cell membrane to the early endosome compartments.

Immunohistochemistry of surgical samples of human ovarian cancer was performed with two different anti-CD206 mAb. Macrophages infiltrating the tumor stroma showed strong reactivity (Figure 2(b)); these results confirmed that the MR is expressed both by ascitic fluid macrophages and by TAM infiltrating solid tumors.

**Figure 3:**
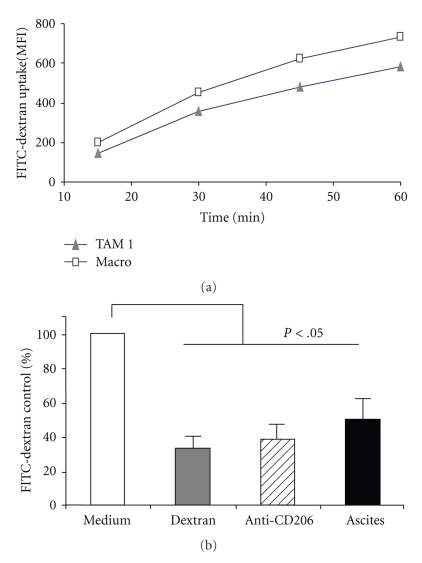
TAMs express a functional endocytic MR. (a) Endocytosis of FITC-Dextran by TAM (triangle) and by in vitro M-CSF-differentiated macrophages (square), evaluated as Mean Fluorescence Intensity (MFI) by flow cytometry. Shown is a representative experiment of 4 performed. (b) FITC-Dextran endocytosis in TAM is significantly inhibited (*P* < .05 Student's *t* tests) by pretreatment with MR-ligands: unconjugated Dextran (1 mg/mL), anti-CD206 mAb (10 ug/mL); and 33%v/v cell-free ascitic fluid from ovarian tumors. Results are expressed as % relative to values of FITC-Dextran uptake in control cells (medium) and are the mean +/− SD of 3 experiments with 3 different TAM preparations.

**Figure 4:**
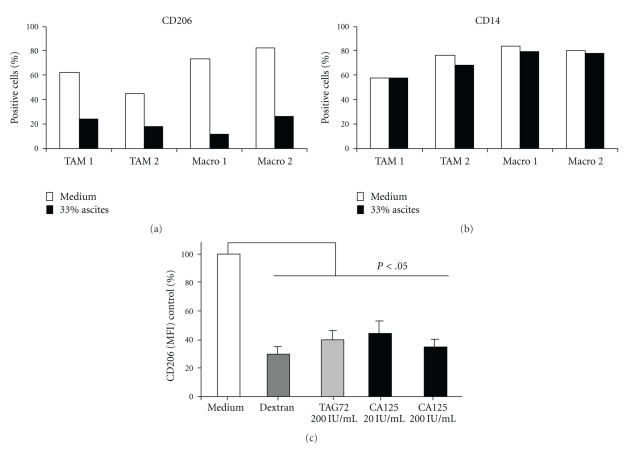
Tumoral mucins induce the internalization of the MR: flow cytometry expression of the MR/CD206 (a) and CD14 (b). Two different TAM preparations (TAM1 and TAM2) and 2 M-CSF-differentiated normal macrophages (Macro1 and Macro2) were pretreated (30 min. room temperature) with 33% v/v ascitic fluid from ovarian tumor patients, prior to staining with anti-CD206 or CD14 mAbs. Results are shown as % of positive cells. (c) Purified TAMs were pretreated with unconjugated Dextran (1 mg/mL); mucin Tag-72 (200 IU/mL); mucin CA125 (20–200 IU/mL) prior to staining with anti-CD206 mAb. Results are shown as % relative to values of Mean fluorescence Intensity (MFI) of CD206 in control cells (medium) and are the mean +/− SD of 4 experiments with 4 different TAM preparations (3 TAM preparations for CA125). *P* < .05 (Student's *t*-tests).

To evaluate MR ability to internalize soluble particles, we incubated TAM with FITC-Dextran, a known ligand of MR. TAM rapidly internalized FITC-Dextran over 60′ period, with a kinetic similar to that of normal macrophages differentiated in vitro with M-CSF (Figure 3(a)). Receptor specificity was checked by pretreating cells with a blocking anti-MR mAb, which resulted in significant inhibition of internalization (Figure 3(b)). Pretreatment of TAM with tumoral ascites (33% v/v) reduced by 50% FITC-Dextran endocytosis, suggesting that ascitic fluids contained putative ligand(s) of the MR. To have further proof of this, we incubated TAM with ascitic fluids prior to staining with anti-CD206 mAb and analyzed in flow cytometry.  Figure 4(a) shows that tumoral ascites did induce the internalization of the MR from the surface of TAM and of normal macrophages: the percentage of surface MR decreased by 60–80% while that of CD14 was unaffected (Figure 4(b)). 

It is established that the MR can bind to MUC1 mucin and to the tumoral mucin TAG-72 [35, 36, 39, 40]. We therefore tested the mucin CA125 that is specifically associated with ovarian cancer. Pretreatment of TAM with TAG-72 or CA125 decreased MR expression, indicating that also CA125 is able to engage the MR and to induce its internalization (Figure 4(c)). 

Notably, both unconjugated Dextran and the other MR ligands tested did not completely block endocytosis or inhibit receptor expression, most likely because—as mentioned above—MR has high recycling ability. In support of this, we noticed that in vitro culture of TAM for 24 hours in the absence of ascitic fluid (i.e., out of the original micro-environment), resulted in higher MR levels compared to TAMs that were immediately tested after isolation (not shown).

### 3.3. Mucin-Mediated Ligation of the MR Modulates Cytokine Production in Human TAM

**Figure 5:**
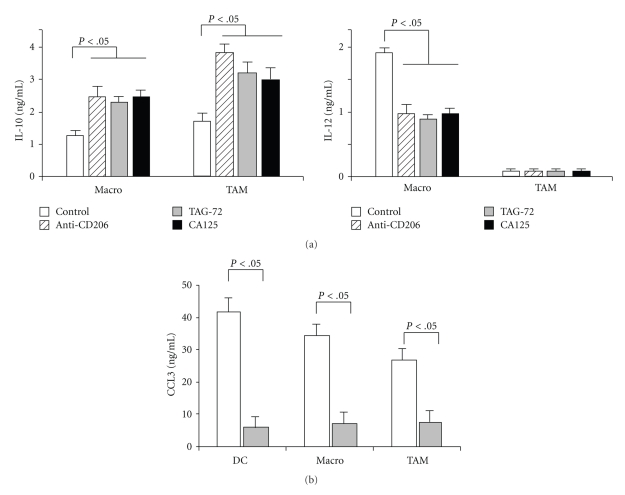
Modulation of cytokine production by tumoral mucin-engagement of the MR. (a) Purified TAM preparations or normal in vitro differentiated macrophages were pretreated (10 min. room temperature) with anti-CD206 (clone WE458, 2 ug/mL), TAG-72 (200 UI/mL), or CA125 (200 UI/mL) prior to stimulation with LPS (1 ug/mL, 24 hrs). Levels of IL-10 (upper panels) and IL-12 (lower panels) were measured in supernatants by ELISA. Results are mean +/− SE of 6 different TAM preparations for IL-10 and 3 for IL-12 (*P* < .05, Student's *t*-tests). (b) Purified TAM, normal in vitro differentiated macrophages, and mono-DC were pretreated (10 min. room temperature) with TAG-72 (200 UI/mL) prior to stimulation with LPS (1 ug/mL, 24 hrs). Levels of CCL3 were measured in supernatants by ELISA. Results are mean +/− SE of 4 different TAM preparations, 3 for macrophages, and mono-DC. *P* < .05 (Student's *t*-tests).

We previously reported in dendritic cells (DCs) that activation of the MR with an agonist mAb or with MUC1 induced a regulatory/immunosuppressive phenotype with a switch of cytokine production characterized by low IL-12 and high IL-10 [27]. Hence, we tested cytokine production of mucin-treated TAM.  Figure 5(a) shows that all tested MR ligands (TAG-72, CA125, and anti-CD206) induced a significant increase of IL-10 in TAM as well as in normal macrophages. By contrast, IL-12 secretion was strongly decreased in normal macrophages (Figure 5(a)). TAMs, as already reported [41], are unable to produce IL-12 even after optimal stimulation with LPS and IFN*γ* (Figure 5(a)). 

Macrophages and DC are a major source of chemokines which importantly amplify the immunological network by recruiting immunocompetent cells at tumor tissues. We investigated the production of the chemokine CCL3, which recruits Th1 and cytotoxic effector lymphocytes.  Figure 5(b) shows that TAG-72 mucin strongly inhibited the secretion of CCL3 by TAM and by in vitro generated macrophages and mono-DC.

Overall these results demonstrate that the MR expressed by TAM recognizes endogenous ligands present in the tumor microenvironment, including the ovarian cancer specific mucin CA125. Mucin-induced MR engagement modulates the cytokine secretion of TAM toward an immune-suppressive phenotype: increase of IL-10, absence of IL-12 and decrease of CCL3. This cytokine profile is likely to contribute to tumor immune escape.

## 4. Discussion

Very little is known about the expression and functional role of CLRs in myeloid cells infiltrating tumors. This study demonstrates that human TAMs express a number of CLRs (e.g., Dectin-1, MDL-1, MGL, DCIR) and most prominently the MR/CD206. Other receptors were not significantly expressed (e.g., DC-SIGN, DEC205, Langerin), in line with their preferential localization on dendritic and Langerhans cells. ENDO180, which shares similarities with the MR, was also poorly represented, and this finding is consistent with its higher expression in fibroblasts [42]. We focused our attention on the MR. Although it has long been known that TAMs bear this receptor—and actually this evidence served as paradigm of their M2-like polarization—no functional characterization of MR-positive TAM has ever been provided. 

We found that both TAMs from solid tumors and those from the ascitic fluid associated to advanced ovarian cancer, have high membrane expression of the MR. Levels of expression were modulated by the tumor microenvironment, as components present in the ascitic fluid were able to induce receptor internalization. 

The MR is one of the oldest CLR described in macrophages [43, 44]. It is an endocytic and phagocytic receptor that binds carbohydrate moieties on several pathogens such as bacteria, fungi, parasites, and viruses and is therefore considered a Pattern Recognition Receptor (PPR). However, it has become increasingly clear that the MR is importantly involved in the silent clearance of circulating inflammatory molecules and degraded matrix components. Mice deficient in the MR do not show increased susceptibility to infections [45, 46] but have elevated levels of lysosomal hydrolases and other glycoproteins which raise up during inflammation and tissue remodelling [47, 48]. These in vivo experiments highlighted its important role in the clearance of unwanted molecules, especially for the MR localized at hepatic sinusoids. 

Not only MR appears to be dispensable for pathogen clearance but also it can negatively modulate pathogen-elicited immune responses. We and others have previously reported that MR-ligation with ManLAM from *M. tubercolosis* or with an agonist anti-MR antibody modulates cytokine production in human DC, with a shift from high to low IL-12, increased IL-10 levels, and defective Th1 immune responses [26, 27]. These results have been confirmed in this study in tumor macrophages activated with agonist anti-MR mAbs. The mechanisms that account for the regulatory functions of the MR are not completely characterized. Unlike other CLRs, MR has no ITIM domain [25]. It has been shown that some CLRs may interfere with TLRs/NF-kB signalling [25, 49], and the MR can indeed physically interact with TLR2 upon internalization [50]. In addition, Pathak et al. reported that mannan induced the upregulation of IRAK-M kinase, which was responsible for the decreased production of proinflammatory cytokines by inhibiting TLR-signaling [51, 52].

 A number of recent studies corroborated the hypothesis that the MR is implicated in the maintenance of homeostasis and tolerance. Macrophages cocultured with mesenchymal stem cells have high MR expression and produce IL-10 [53]. Royer et al. reported that allergens inducing Th2-polarized responses express MR-binding carbohydrate moieties; the receptor contributed to T cell polarization as its silencing in DC strongly impaired Th2 development [54]. Macrophages localized at sites where inflammation could be particularly harmful are usually strongly MR-positive (e.g., alveolar macrophages and brain microglia). Further, at the maternal-foetal interface the presence of immune cells is important to preserve tolerance as well as for active remodelling of uterine vessels. Decidual macrophages are a major source of IL-10 and IDO [55] and express high levels of the MR [40]. MR recognizes several endogenous ligands and acts as a bridge between innate immunity and homeostasis [25, 56]. For instance, circulating hormones, like lutropin and thyrotropin are bound by the MR cystein-rich domain [57]. Collagen is another MR-ligand and the receptor may serve important scavenger functions [58]. 

 In the context of a tumor microenvironment, where highly glycosylated molecules such as mucins are present [59] CLRs encounter several putative ligands. MGL and DC-SIGN recognize cancer-specific glycosylation changes of the mucin MUC1, in particular the carbohydrate sialyl Lewis^X^ and the sialyl TN epitope [60]; MUC1 and TAG-72 bind also the MR [27, 35, 36]. We previously reported that mono-DCs differentiated in the presence of tumor cell-derived mucins have a tolerogenic/regulatory cytokine profile [34]. In the present study we extended this observation to tumor macrophages: TAMs bound and internalized both TAG-72 and the ovarian cancer-associated mucin CA125 via the MR, indicating a specific recognition by this receptor. Further, these mucins interfered with the LPS-induced production of IL-10 and of the chemokine CCL3. These results are in line with the observation that another tumoral mucin, the carcinoembryonic antigen (CEA) highly expressed by colon cancer cells, binds DC-SIGN on DC and induces increased secretion of IL-10 and IL-6 [61]. Hence, evidence is accumulating that CLR recognition of tumor glycans leads to the expression of the potent immunoregulatory cytokine IL-10. In the tumor microenvironment IL-10 has detrimental effects on immune responses as it promotes the polarization of M2 macrophages inhibits the differentiation of Th1 lymphocytes while favouring that of Treg [62]. Interestingly, a recent study showed that distinct TAM subsets can be distinguished on the basis of differential expression of MHC II molecules. TAMs characterized by MHC II^low^ and suppressive activity on T cell proliferation have higher expression of the MR [63].

In addition, MR is expressed by endothelial cells of lymphatic vessels [64] and it has been demonstrated to be implicated in the dissemination of tumor cells along lymphatics [33]. Recently, Arteta et al. reported that MR-positive liver sinusoidal vessels also support hepatic metastasis of colon cancer cells by a mechanism that involves IL-1-induced upregulation of the MR [65].

 Thus, while under physiological conditions the regulatory effect of CLRs on innate immunity cells is finalized to the preservation of homeostasis, in pathological conditions such as cancer, CLR activity may hamper the activation of a protective immune response and actually favour tumor spread.

In conclusion, we have demonstrated that the MR on human TAM can be engaged by mucins present in the tumor microenvironment. This interaction further enhances their immunosuppressive phenotype and can be considered as another mechanism of tumor immune evasion.

## Figures and Tables

**Table 1:** Affymetrix Gene Expression analysis of selected C-type lectin receptors in human Tumor-Associated Macrophages (TAMs).

Gene symbol/ other names	Intensity**°**	Modulation^§^ LPS/IFN*γ*	Modulation^§^ IL-10
MRC1/CD206 CLEC13D	562 ± 76	0,1*	1,5*
Dectin-1 CLEC7A	482 ± 63	0,3*	1,4*
MDL-1 CLEC5A	468 ± 94	0,3*	0,7
DCIR CLEC4A	359 ± 37	0,7	1,1
MGL-1/CD301 CLEC10A	258 ± 85	0,9	1,1
DCL-1/CD302 CLEC13A	160 ± 42	0.9	1
ENDO-180/CD280 CLEC13E	111 ± 72	0,8	1
DEC-205/CD205 CLEC13B	77 ± 42	2,9*	0,7
DC-SIGN/CD209 CLEC4L	40 ± 13	0,8	0,9
Langerin/ CD207 CLEC4K	42 ± 11	0,9	0,9
PLA2R CLEC13C	25 ± 6	1	1

**°**Normalized intensity, **^§^**Fold over untreated TAM. Results are expressed as median values ± SE of 7 different TAM preparations and are presented as normalized intensity signals: modulation of CLR genes after TAM treatment with LPS/IFN*γ* or IL-10 for 18 hrs. Results are presented as fold over untreated TAM, *P < .05 versus untreated. Experiment of CLR modulation by cytokines was performed in 4 TAM preparations.

## References

[1] Allavena P, Sica A, Garlanda C, Mantovani A (2008). The Yin-Yang of tumor-associated macrophages in neoplastic progression and immune surveillance. *Immunological Reviews*.

[2] Solinas G, Germano G, Mantovani A, Allavena P (2009). Tumor-associated macrophages (TAM) as major players of the cancer-related inflammation. *Journal of Leukocyte Biology*.

[3] Lewis CE, Pollard JW (2006). Distinct role of macrophages in different tumor microenvironments. *Cancer Research*.

[4] Galon J, Costes A, Sanchez-Cabo F (2006). Type, density, and location of immune cells within human colorectal tumors predict clinical outcome. *Science*.

[5] Laghi L, Bianchi P, Miranda E (2009). CD3+ cells at the invasive margin of deeply invading (pT3-T4) colorectal cancer and risk of post-surgical metastasis: a longitudinal study. *The Lancet Oncology*.

[6] Bingle L, Brown NJ, Lewis CE (2002). The role of tumour-associated macrophages in tumour progression: implications for new anticancer therapies. *Journal of Pathology*.

[7] Pollard JW (2004). Tumour-educated macrophages promote tumour progression and metastasis. *Nature Reviews Cancer*.

[8] Condeelis J, Pollard JW (2006). Macrophages: obligate partners for tumor cell migration, invasion, and metastasis. *Cell*.

[9] Mantovani A, Allavena P, Sica A, Balkwill F (2008). Cancer-related inflammation. *Nature*.

[10] Joyce JA, Pollard JW (2009). Microenvironmental regulation of metastasis. *Nature Reviews Cancer*.

[11] Mantovani A, Sozzani S, Locati M, Allavena P, Sica A (2002). Macrophage polarization: tumor-associated macrophages as a paradigm for polarized M2 mononuclear phagocytes. *Trends in Immunology*.

[12] Gordon S, Taylor PR (2005). Monocyte and macrophage heterogeneity. *Nature Reviews Immunology*.

[13] Martinez FO, Helming L, Gordon S (2009). Alternative activation of macrophages: an immunologic functional perspective. *Annual Review of Immunology*.

[14] Goerdt S, Orfanos CE (1999). Other functions, other genes: alternative activation of antigen- presenting cells. *Immunity*.

[15] Mantovani A, Sica A, Locati M (2005). Macrophage polarization comes of age. *Immunity*.

[16] Talmadge JE, Donkor M, Scholar E (2007). Inflammatory cell infiltration of tumors: Jekyll or Hyde. *Cancer and Metastasis Reviews*.

[17] Steidl C, Lee T, Shah SP (2010). Tumor-associated macrophages and survival in classic Hodgkin’s lymphoma. *The New England Journal of Medicine*.

[18] Zumsteg A, Christofori G (2009). Corrupt policemen: inflammatory cells promote tumor angiogenesis. *Current Opinion in Oncology*.

[19] Weis WI, Taylor ME, Drickamer K (1998). The C-type lectin superfamily in the immune system. *Immunological Reviews*.

[20] McGreal EP, Miller JL, Gordon S (2005). Ligand recognition by antigen-presenting cell C-type lectin receptors. *Current Opinion in Immunology*.

[21] Janeway CA, Medzhitov R (2002). Innate immune recognition. *Annual Review of Immunology*.

[22] Robinson MJ, Sancho D, Slack EC, LeibundGut-Landmann S, Sousa CR (2006). Myeloid C-type lectins in innate immunity. *Nature Immunology*.

[23] Geijtenbeek TBH, van Vliet SJ, Engering A, ’T Hart BA, van Kooyk Y (2004). Self- and nonself-recognition by C-type lectins on dendritic cells. *Annual Review of Immunology*.

[24] García-Vallejo JJ, Van Kooyk Y (2009). Endogenous ligands for C-type lectin receptors: the true regulators of immune homeostasis. *Immunological Reviews*.

[25] Geijtenbeek TBH, Gringhuis SI (2009). Signalling through C-type lectin receptors: shaping immune responses. *Nature Reviews Immunology*.

[26] Nigou J, Zelle-Rieser C, Gilleron M, Thurnher M, Puzo G (2001). Mannosylated lipoarabinomannans inhibit IL-12 production by human dendritic cells: evidence for a negative signal delivered through the mannose receptor. *Journal of Immunology*.

[27] Chieppa M, Bianchi G, Doni A (2003). Cross-linking of the mannose receptor on monocyte-derived dendritic cells activates an anti-inflammatory immunosuppressive program. *Journal of Immunology*.

[28] Geijtenbeek TBH, Van Vliet SJ, Koppel EA (2003). Mycobacteria target DC-SIGN to suppress dendritic cell function. *Journal of Experimental Medicine*.

[29] Dzionek A, Sohma Y, Nagafune J (2001). BDCA-2, a novel plasmacytoid dendritic cell-specific type II C-type lectin, mediates antigen capture and is a potent inhibitor of interferon *α*/*β* induction. *Journal of Experimental Medicine*.

[30] Meyer-Wentrup F, Benitez-Ribas D, Tacken PJ (2008). Targeting DCIR on human plasmacytoid dendritic cells results in antigen presentation and inhibits IFN-*α* production. *Blood*.

[31] Fujikado N, Saijo S, Yonezawa T (2008). Dcir deficiency causes development of autoimmune diseases in mice due to excess expansion of dendritic cells. *Nature Medicine*.

[32] van Vliet SJ, Gringhuis SI, Geijtenbeek TBH, van Kooyk Y (2006). Regulation of effector T cells by antigen-presenting cells via interaction of the C-type lectin MGL with CD45. *Nature Immunology*.

[33] Marttila-Ichihara F, Turja R, Miiluniemi M (2008). Macrophage mannose receptor on lymphatics controls cell trafficking. *Blood*.

[34] Monti P, Leone BE, Zerbi A (2004). Tumor-derived MUC1 mucins interact with differentiating monocytes and induce IL-10IL-12 regulatory dendritic cell. *Journal of Immunology*.

[35] Hiltbold EM, Vlad AM, Ciborowski P, Watkins SC, Finn OJ (2000). The mechanism of unresponsiveness to circulating tumor antigen MUC1 is a block in intracellular sorting and processing by dendritic cells. *Journal of Immunology*.

[36] Rughetti A, Pellicciotta I, Biffoni M (2005). Recombinant tumor-associated MUC1 glycoprotein impairs the differentiation and function of dendritic cells. *Journal of Immunology*.

[37] Solinas G, Schiarea S, Liguori M (2010). Tumor-conditioned macrophages secrete migration-stimulating factor: a new marker for M2-polarization, influencing tumor cell motility. *Journal of Immunology*.

[38] Saccani A, Schioppa T, Porta C (2006). p50 nuclear factor-*κ*B overexpression in tumor-associated macrophages inhibits M1 inflammatory responses and antitumor resistance. *Cancer Research*.

[39] Apostolopoulos V, Barnes N, Pietersz GA, McKenzie IFC (2000). Ex vivo targeting of the macrophage mannose receptor generates anti-tumor CTL responses. *Vaccine*.

[40] Laskarin G, Cupurdija K, Sotosek Tokmadzic V (2005). The presence of functional mannose receptor on macrophages at the maternal-fetal interface. *Human Reproduction*.

[41] Sica A, Saccani A, Bottazzi B (2000). Autocrine production of IL-10 mediates defective IL-12 production and NF-*κ*B activation in tumor-associated macrophages. *Journal of Immunology*.

[42] Thomas EK, Nakamura M, Wienke D, Isacke CM, Pozzi A, Liang P (2005). Endo180 binds to the C-terminal region of type I collagen. *The Journal of Biological Chemistry*.

[43] Ezekowitz RA, Gordon S (1982). Surface properties of activated macrophages: sensitized lymphocytes, specific antigen and lymphokines reduce expression of antigen F4/80 and FC and mannose/fucosyl receptors, but induce Ia. *Advances in Experimental Medicine and Biology*.

[44] Tietze C, Schlesinger P, Stahl P (1982). Mannose-specific endocytosis receptor of alveolar macrophages: demonstration of two functionally distinct intracellular pools of receptor and their roles in receptor recycling. *Journal of Cell Biology*.

[45] Swain SD, Lee SJ, Nussenzweig MC, Harmsen AG (2003). Absence of the macrophage mannose receptor in mice does not increase susceptibility to pneumocystis carinii infection in vivo. *Infection and Immunity*.

[46] Lee SJ, Zheng NY, Clavijo M, Nussenzweig MC (2003). Normal host defense during systemic candidiasis in mannose receptor-deficient mice. *Infection and Immunity*.

[47] Lee SJ, Evers S, Roeder D (2002). Mannose receptor-mediated regulation of serum glycoprotein homeostasis. *Science*.

[48] Mi Y, Shapiro SD, Baenziger JU (2002). Regulation of lutropin circulatory half-life by the mannose/N-acetylgalactosamine-4-SO_4_ receptor is critical for implantation in vivo. *Journal of Clinical Investigation*.

[49] Harnett W, Harnett MM (2010). Helminth-derived immunomodulators: can understanding the worm produce the pill?. *Nature Reviews Immunology*.

[50] Tachado SD, Zhang J, Zhu J, Patel N, Cushion M, Koziel H (2007). Pneumocystis-mediated IL-8 release by macrophages requires coexpression of mannose receptors and TLR2. *Journal of Leukocyte Biology*.

[51] Pathak SK, Basu S, Bhattacharyya A, Pathak S, Kundu M, Basu J (2005). Mycobacterium tuberculosis lipoarabinomannan-mediated IRAK-M induction negatively regulates toll-like receptor-dependent interleukin-12 p40 production in macrophages. *The Journal of Biological Chemistry*.

[52] Zhang J, Tachado SD, Patel N (2005). Negative regulatory role of mannose receptors on human alveolar macrophage proinflammatory cytokine release in vitro. *Journal of Leukocyte Biology*.

[53] Kim J, Hematti P (2009). Mesenchymal stem cell-educated macrophages: a novel type of alternatively activated macrophages. *Experimental Hematology*.

[54] Royer P-J, Emara M, Yang C (2010). The mannose receptor mediates the uptake of diverse native allergens by dendritic cells and determines allergen-induced T cell polarization through modulation of IDO Activity. *Journal of Immunology*.

[55] Nagamatsu T, Schust DJ (2010). The immunomodulatory roles of macrophages at the maternal-fetal interface. *Reproductive Sciences*.

[56] Gazi U, Martinez-Pomares L (2009). Influence of the mannose receptor in host immune responses. *Immunobiology*.

[57] Leteux C, Chai W, Loveless RW (2000). The cysteine-rich domain of the macrophage mannose receptor is a multispecific lectin that recognizes chondroitin sulfates A and B and sulfated oligosaccharides of blood group Lewis and Lewis(x) types in addition to the sulfated N-glycans of lutropin. *Journal of Experimental Medicine*.

[58] Martinez-Pomares L, Wienke D, Stallion R (2006). Carbohydrate-independent recognition of collagens by the macrophage mannose receptor. *European Journal of Immunology*.

[59] Kufe DW (2009). Mucins in cancer: function, prognosis and therapy. *Nature Reviews Cancer*.

[60] Aarnoudse CA, Vallejo JJG, Saeland E, Van Kooyk Y (2006). Recognition of tumor glycans by antigen-presenting cells. *Current Opinion in Immunology*.

[61] Nonaka M, Ma BY, Murai R (2008). Glycosylation-dependent interactions of C-type lectin DC-SIGN with colorectal tumor-associated Lewis glycans impair the function and differentiation of monocyte-derived dendritic cells. *Journal of Immunology*.

[62] O’Garra A, Vieira P (2004). Regulatory T cells and mechanisms of immune system control. *Nature Medicine*.

[63] Movahedi K, Laoui D, Gysemans C (2010). Different tumor microenvironments contain functionally distinct subsets of macrophages derived from Ly6C(high) monocytes. *Cancer Research*.

[64] Irjala H, Alanen K, Grénman R, Heikkilä P, Joensuu H, Jalkanen S (2003). Mannose receptor (MR) and common lymphatic endothelial and vascular endothelial receptor (CLEVER)-1 direct the binding of cancer cells to the lymph vessel endothelium. *Cancer Research*.

[65] Arteta B, Lasuen N, Lopategi A, Sveinbjörnsson B, Smedsrød B, Vidal-Vanaclocha F (2010). Colon carcinoma cell interaction with liver sinusoidal endothelium inhibits organ-specific antitumor immunity through interleukin-1-induced mannose receptor in mice. *Hepatology*.

